# Supplementary figures and images for: Color for the perceptual organization of the pictorial plane: Victor Vasarely's legacy to Gestalt psychology
Source: Heliyon. 2020 Jul 16;6(7):e04375. doi: 10.1016/j.heliyon.2020.e04375 (PMC7365985; doi:10.1016/j.heliyon.2020.e04375)

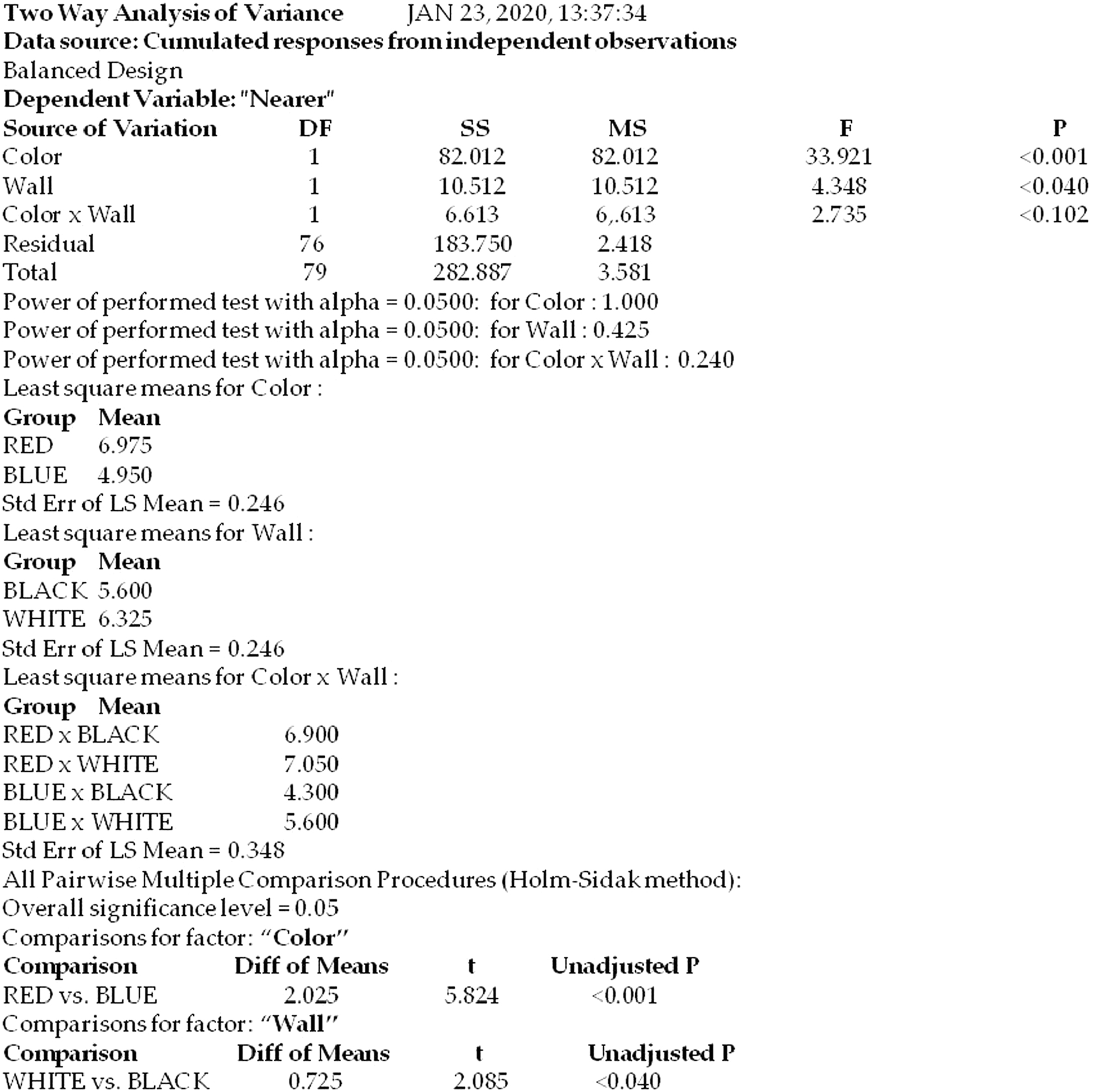

Supplement: TABLE-S1 [file figs1.jpg]

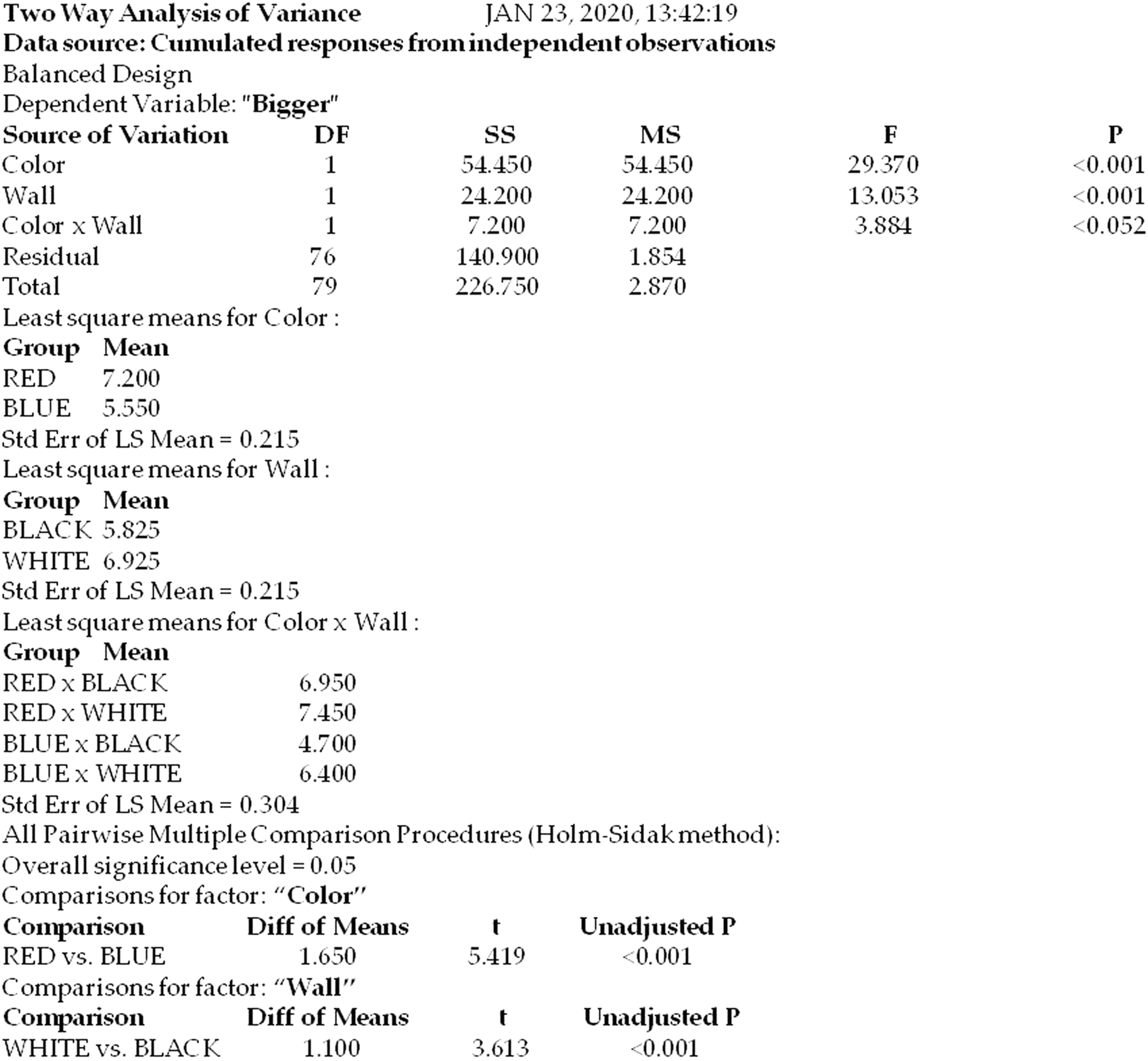

Supplement: TABLE-S2 [file figs2.jpg]

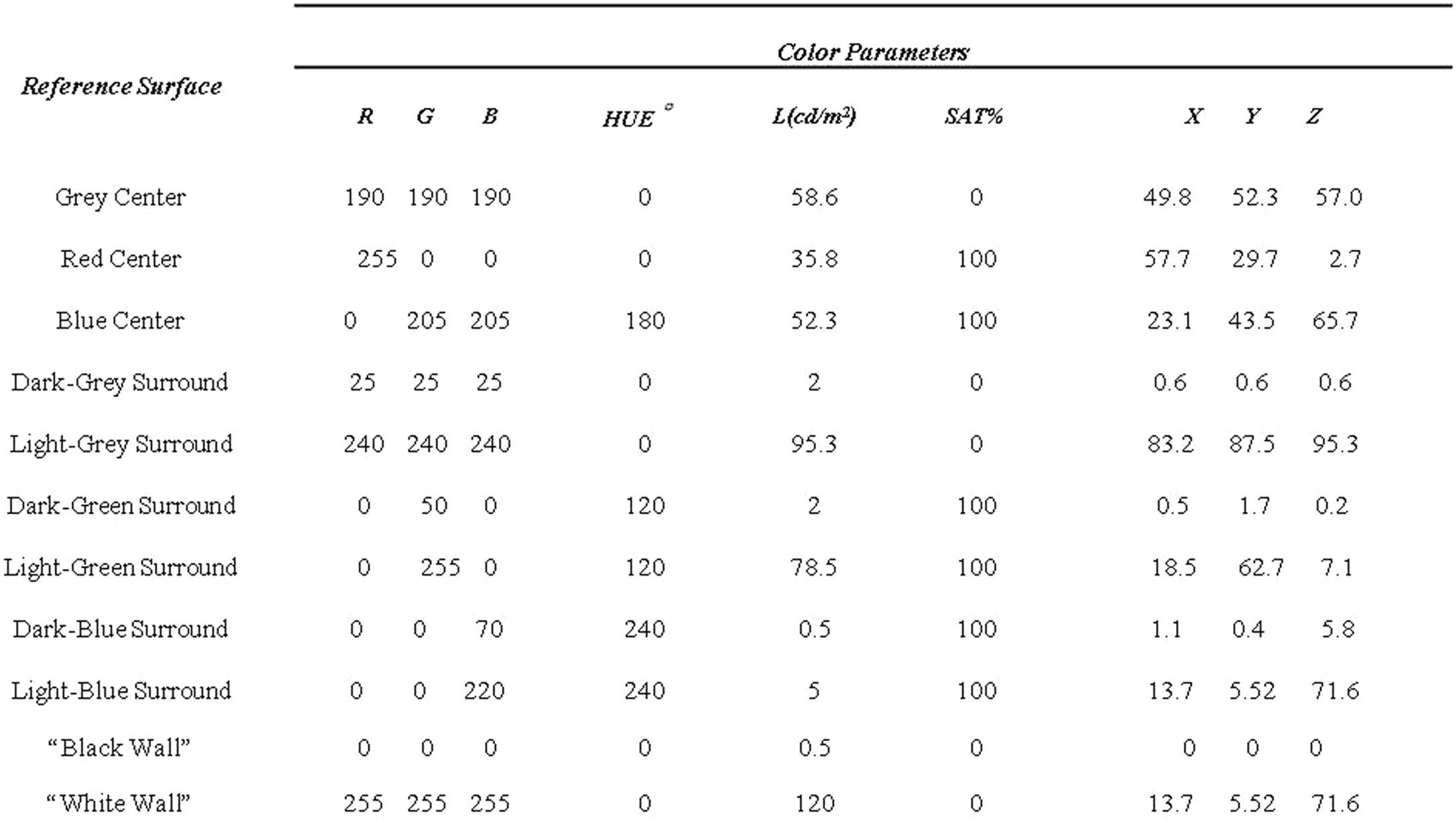

Supplement: TABLE-S3 [file figs3.jpg]
